# Unlocking an acute psychiatric ward: the impact on unauthorised absences, assaults and seclusions

**DOI:** 10.1192/pb.bp.115.052944

**Published:** 2017-04

**Authors:** Ben Beaglehole, John Beveridge, Warren Campbell-Trotter, Chris Frampton

**Affiliations:** 1 Department of Psychological Medicine, University of Otago, Christchurch, New Zealand; 2 Canterbury District Health Board, Christchurch, New Zealand

## Abstract

**Aims and method** The acute psychiatric in-patient service in Christchurch, New Zealand, recently changed from two locked and two unlocked wards to four open wards. This provided the opportunity to evaluate whether shifting to an unlocked environment was associated with higher rates of adverse events, including unauthorised absences, violent incidents and seclusion. We compared long-term adverse event data before and after ward configuration change.

**Results** Rates of unauthorised absences increased by 58% after the change in ward configuration (*P* = 0.005), but seclusion hours dropped by 53% (*P* = 0.001). A small increase in violent incidents was recorded but this was not statistically significant.

**Clinical implications** Although unauthorised absences increased, the absence of statistically significant changes for violent incidents and a reduction in seclusion hours suggest that the change to a less restrictive environment may have some positive effects.

The locking of psychiatric institutions is an important topic because detention involves significant restrictions on human rights, ensconced in the Universal Declaration of Human Rights. However, studies that have evaluated the impact of unlocking a psychiatric ward are rare; we were only able to identify four such studies spanning 7-decade-long assessing in this area. Lang *et al*^1^ evaluated a policy change that resulted in a ward changing from being largely locked for 6 months to being largely open for 6 months and found that negative outcomes were more common during the closed time period. Other studies are considerably older^2,3^ and were largely positive in their findings, with the exception of the study by Molnar *et al*,^4^ which reported an increase in the frequency of ‘elopement’ (unauthorised absence) from 2.5 to 7% of admissions in the context of changing ward policy to allow a largely unlocked environment.

Any consideration of locked psychiatric wards must be viewed in the context of emerging policy that recommends minimising the use of locked psychiatric wards^5^ and emphasises a duty to provide psychiatric care in the least restrictive environment. A desire to provide least restrictive care created the impetus for our local psychiatric service to shift from an acute in-patient environment consisting of two locked wards and two unlocked wards to four largely unlocked wards. It also provided the opportunity to evaluate the impact of the change through reporting of long-term data detailing adverse events, such as unauthorised absences, ward violence and rates of seclusion, prior to and following the changes in ward environment.

## Method

Data for this study were taken from the acute in-patient service in Christchurch, New Zealand. The service is the sole provider of acute in-patient services to adults aged 18–65 in the city of Christchurch, outlying towns and rural areas, with the exception of forensic patients, patients with an intellectual disability and in-patients requiring planned detoxification from substances, who are admitted elsewhere. The acute in-patient service receives admissions from community and emergency psychiatric services when care in the community is no longer feasible, with a typical case mix consisting of patients with affective disorders, psychotic disorders and personality disorders.

**Fig. 1:**
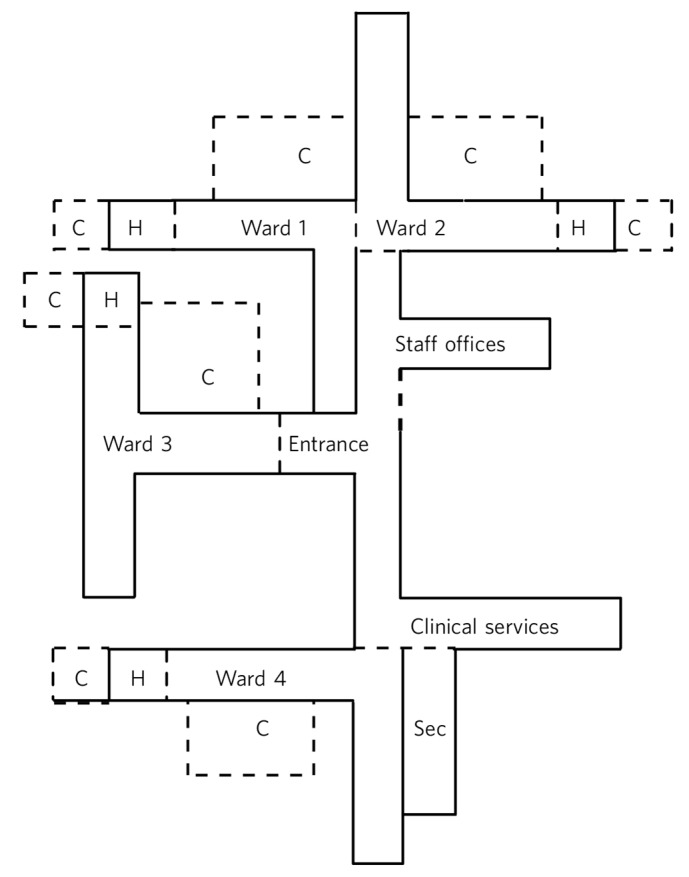
Acute in-patient service: ward layout. H, high care area; Sec, seclusion area; C, courtyard.

The service underwent major architectural change in 2013. Before, the service was configured with two locked wards (totalling 20 beds), two wards (totalling 44 beds) that were predominantly unlocked except overnight, and a seclusion area with 3 seclusion rooms. In response to service development initiatives and the desire to provide less restrictive care, the wards were reconfigured into 4 largely unlocked 16-bed wards (except overnight when the wards remain locked). At the end of each ward is a zone called the ‘high care area’, which is quieter than the general ward and provides care for up to three of the more unwell patients. Each high care area is continuous with its adjacent ward and can be designated ‘high care’ when doors are closed or locked, or be regarded as part of the general ward when extra clinical input is not required. The décor and bedrooms of the high care area are identical to the adjacent ward, but an additional lounge, bathroom and courtyard are provided in order for the high care area to act as a self-sufficient unit when required. All wards, including the high care areas, are intended to be unlocked as much as possible but each ward or high care area can be locked separately in order to detain a patient if this is deemed necessary. The high care area bedrooms were not outfitted for seclusion but the seclusion area remained unaltered and could still be used if required. Figure 1 presents details of the ward layout. If the ward or a high care area is locked, it is recorded in the restraint register as an environment restraint, and is therefore available for review.

In addition to these structural changes, nursing staff numbers increased, from 27 nurses routinely rostered on the wards to 34 nurses. However, if a nurse was required to provide one-to-one supervision after the change, this needed to occur within existing staff levels, whereas previously this was not the case. The frequency of one-to-one supervision pre- and post-change was not systematically recorded and is not readily available for review.

The acute in-patient service routinely records data on unauthorised absences, seclusions and violent incidents. Unauthorised absences include patients who are under involuntary mental health legislation and leave the ward without permission or fail to return from authorised leave, and voluntary patients who leave the ward outside of their agreed treatment parameters or fail to return from agreed leave. Nursing staff follow procedures when absences occur, including filling out a form documenting the absence which is then collated in a central database.

Violent incidents are assessed clinically and categorised into a number of subcategories including verbal abuse, threats of violence and assaults; all are recorded and collated centrally.

Seclusion is initiated by nursing staff as an intervention of last resort for managing a situation of imminent or actual violence. In response to international and New Zealand initiatives,^6^ seclusion reduction initiatives became embedded into the routine care environment of the acute in-patient service from 2010 and the goal of reducing seclusion occurred alongside the plan to provide a less restrictive environment through the ward redevelopment. Although seclusion rates are likely to be reduced by the seclusion reduction initiatives, the change in ward environment had the potential to hinder or assist the goal of reducing seclusion. As a consequence, we report seclusion rates which are collated centrally on a database and are therefore available for review.

The AWOL (absent without leave), violent incidents and seclusion databases have all collected data over an extended period. In de-identified form, they provided the opportunity to examine rates of unauthorised absences, seclusions and violent incidents for 18 months prior to the change in ward configuration and compare this to 18 months following the change. The mean rates and standard deviations of the monthly adverse events were calculated for the pre- and post-change 18-month intervals. As the data were non-parametric in nature, the Mann-Whitney *U*-test was used to compare the 18 months prior to the change with the 18 months following the change. The change in ward environment was staggered during June and July 2013; this time period was therefore not included in any data analysis as our goal was to examine a longer-term impact of the policy change as opposed to short-term effects during and following the transition.

We also report frequency and duration of the use of environmental restraint to clarify whether or not the intended change to a largely unlocked environment was successful or resulted in frequent locking of the newly configured wards. The frequency of Safety Assessment Code (SAC) 1 and 2 incidents (adverse events involving serious, major or extreme harm to patients) involving psychiatric in-patients is also provided pre- and post-change and examined for the possibility of a change in more serious adverse events that would not be detected by previous methods.

Ward occupancy data are routinely collected by means of a census taken at midnight every 24 hours. Admission data to the acute in-patient service and the forensic service are also recorded daily. Forensic in-patients are predominantly admitted from forensic out-patient services, courts and prisons, and not from general out-patient services. However, we examined admission and occupancy data for these services to test for the possibility that changes in outcome data were confounded by variations in occupancy or transfer to the more secure environment of the forensic service. The rate of monthly admissions pre- and post-change was compared using the Mann-Whitney *U*-test.

Although this was a study of group data and individual files were not accessed, ethics approval was sought and granted by the local University of Otago ethics committee (reference number HD 14/21).

## Results

**Fig. 2:**
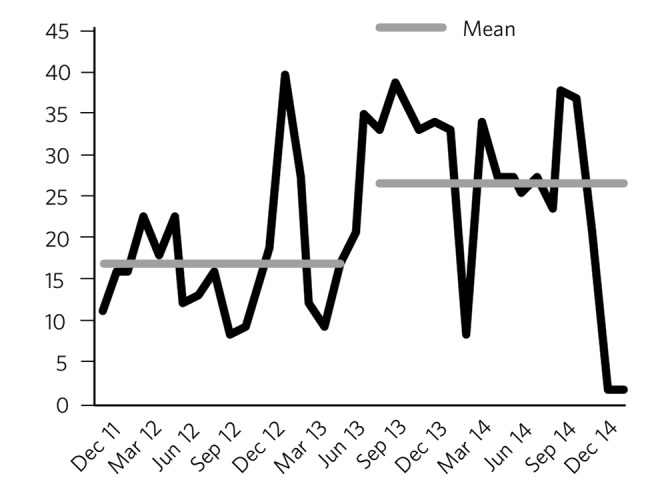
Unauthorised absences before and after the change in ward configuration.

Longitudinal data on monthly unauthorised absences are presented in Fig. 2. The mean rate for the 18 months prior to the new ward configuration (December 2011-May 2013) was 16.9 (s.d. = 7.7) compared with 26.7 (s.d. = 11.2) for 18 months following the change in ward configuration (August 2013-January 2015). This represented a mean increase of 9.7 unauthorised absences per month and a percentage increase of 58% that was statistically significant (*P* = 0.005). As some literature suggests unauthorised absences may be seasonal, means were also calculated for the year pre- and post-change to ensure identical calendar months were compared, and the results were similar (16.6 (s.d. = 9.1) *v.* 29.6 (s.d. = 7.5), respectively).

**Fig. 3:**
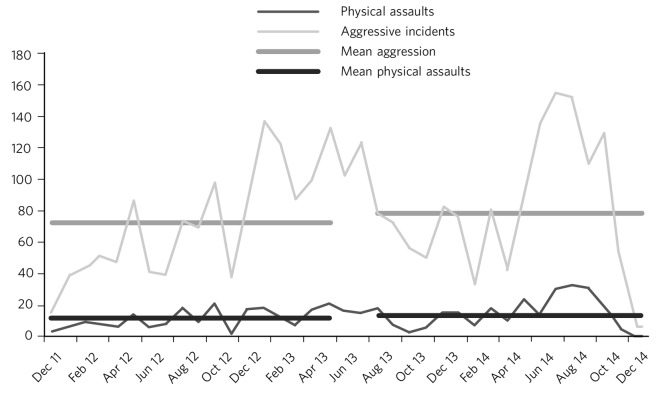
Aggressive incidents and physical assaults before and after the change in ward configuration.

Violent incidents were examined through the extraction of data recorded under the category of aggression, which includes verbal abuse, verbal threats, physical threats and physical assaults. Specific data were also extracted on physical assaults to assess for more significant violence. Figure 3 shows the longitudinal data for all violent incidents and the mean monthly rates for 18 months pre- and 18 months post-ward changes, which were 72.3 (s.d. = 34.5) and 78.2 (s.d. = 43.1), respectively. This represented a mean increase of 5.9 violent incidents/month, or an 8% increase in incidents, which was not statistically significant (*P* = 0.696). With regard to physical assaults, the mean difference of 2.8 assaults/month, from 11.5 (s.d. = 5.9) before to 14.3 (s.d. = 10.1) after, was also not statistically significant (*P* = 0.628) (Fig. 3).

**Fig. 4:**
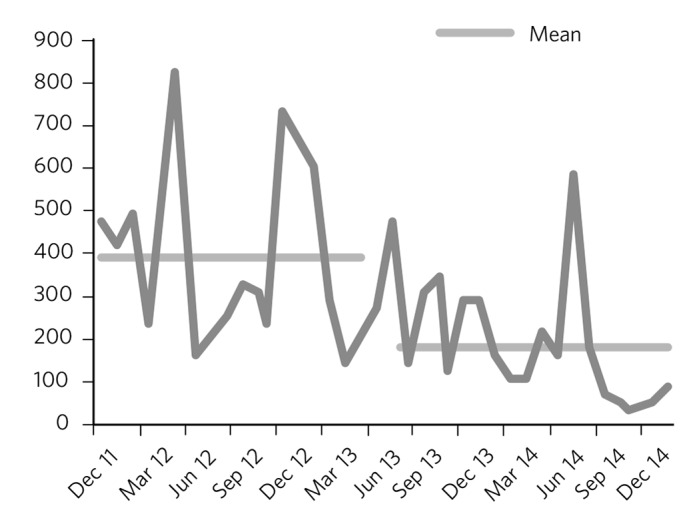
Seclusion hours before and after the change in ward configuration.

Another assessed variable was the longitudinal data on monthly seclusion hours over the study period as well as the mean rates of monthly seclusion (hours/month) (Fig. 4). The mean length of seclusion prior to the change was 391.5 (s.d. 203.0) compared with 185.2 (s.d. 135.6) following the change. This represented a mean drop of 206 hours/month or a percentage drop of 53% that was statistically significant (*P* = 0.001).

Occupancy was recorded according to bed nights/month and converted to a percentage of available bed nights. Occupancy varied between 80 and 101%, with a mean occupancy of 91% over the study period. It is likely that the single month that experienced more than 100% occupancy was very busy and included extra persons in rooms (e.g. partners, who do not routinely stay) being entered in the census data. Percentage occupancy data were largely stable over the study period and were without systematic trends that could account for the significant increases in unauthorised absences or significant reduction in seclusion hours. In keeping with the occupancy data, the rate of new admissions to the acute in-patient service was also largely stable over the study period (mean monthly admissions for the 18 months pre-change 100.4 compared with 107.8 for the 18 months post-change, *P*=0.071). The mean number of monthly admissions to the forensic service increased from 3.1 before the new ward configuration to 4.4 after; however, the increase was not caused by in-patients from the acute in-patient service as a total of 3 patients were transferred from the acute in-patient service to the forensic service for the 18 months prior to the ward change compared with 4 patients in the 18 months following the change.

The frequency of environmental restraint (locking of the high care areas or whole wards) revealed that, on average, there were 16 times per month when either a high care area or a whole ward was locked following the change in ward configuration. This meant that on average, each of the four wards had 4 periods each month in which part or the whole of the ward was locked. The frequency of locked periods/month varied from 0 for some wards to a maximum of 19, when the high care area of one ward was locked on multiple occasions in response to a single patient's multiple attempts to leave. In 37% of the environmental restraints, just the high care areas were locked as opposed to whole wards (affecting only 1–3 patients at a time). The mean time an area was locked was 120 minutes, and the range varied from 1 minute to 920 minutes. These results can be compared with the period prior to the new ward configuration, which had two wards (affecting up to 20 patients) constantly locked and occasional periods when the two open wards were locked in response to clinical pressure.

The total number of SAC1 and SAC2 events involving in-patients was 14 over the study period. Of these, 10 occurred before the change in ward configuration, 0 occurred during the transition period, and 4 occurred after the changes were made. For SAC1 incidents resulting in the death of a patient, 7 occurred prior to the ward changes (4 deaths occurred on the ward, 2 while patients were on granted leave, and 1 while on unauthorised absence from the ward) and 4 deaths occurred following the ward changes (3 on the ward and 1 while on granted leave). No SAC2 incidents occurred for patients who were on leave in the community or during an unauthorised absence.

## Discussion

This study examined a range of adverse indicators over an extended time period in order to clarify whether or not a change in ward environment from two locked and two unlocked wards to a largely unlocked environment was associated with an increase in adverse events. The principal finding was that a significant increase in unauthorised absences occurred. However, significant decreases in the use of seclusion and non-significant increases in violent outcomes were also observed, although the reduction in seclusion occurred alongside national and local initiatives to minimise the use of seclusion.

The new ward configuration still made provision for the locking of wards. However, this was only done for relatively short periods (mean 120 min) and less often than prior to the ward redevelopment, when 2 wards affecting 20 patients were continuously locked. In addition, in 37% of the occasions when locking occurred, only a high care area was locked, meaning that 3 or fewer patients were affected. These numbers suggest that the unlocking of two wards and the change in ward environment did not result in wide-spread or prolonged locking of the new ward configuration.

The occupancy and admission data were without systematic trends to explain the changes noted. In addition, there was minimal flow of patients from the acute in-patient service to the forensic service, suggesting that more difficult patients who may be over-represented in adverse incident data were not transferred to forensic services after the wards were reconfigured.

Previous studies of unauthorised absences from psychiatric wards have raised concerns about rare but serious adverse events that have occurred while patients are absent.^7^ Although the low base rate of these events makes analysis difficult, it is reassuring for those considering a transition to the provision of a largely unlocked environment that of the 14 SAC1 and SAC2 events occurring over the study period, 10 occurred prior to the ward change and only 4 occurred afterwards. In addition, of the adverse events resulting in the death of a patient, 7 occurred before the ward changes and 4 afterwards. Although total numbers of unauthorised absences increased, there was only one death involving a patient who had left the ward without being granted leave, and it occurred prior to the ward change. All other community deaths over the study period involving in-patients occurred for patients who had been granted leave.

### Strengths and limitations

One strength of the study was the routine collection of outcome data by hospital staff who were unaware that the data would later be used for study purposes. As a consequence, changes in reporting behaviour could not arise as a result of study influences because of the retrospective nature of the study conception and design. A further strength is the longitudinal nature of the data-set that allowed us to make before-and-after comparisons and consider longer-term effects, as opposed to solely focusing on the transition period during which staff are adapting to changes. These longer-term effects were thought to be more important in evaluating the impact of the change in environment and can be taken into account by service leaders in other locations considering similar changes. Although the longer-term outcomes were our primary area of interest, it is also reassuring that the transition period did not coincide with any SAC1 or SAC2 events, or a spike in the other adverse events evaluated by the study.

Our main limitation was that the study design was not experimental in nature. As the study was uncontrolled, our methodology allows comments to be made on associations between adverse events and the ward changes, but demonstrating causation is not possible. In particular, there was a service initiative to reduce seclusion that started prior to the study period. There were also increases in the numbers of routinely rostered nursing staff on the acute in-patient service after the ward change. This means that the relative influences of the change in ward configuration, the seclusion-reducing initiatives and the changes in nursing numbers on the adverse event rates are hard to quantify. It is therefore possible that increases in seclusion might have been observed if the changes in ward configuration had occurred in isolation. However, it is also reassuring to note that no such increases were seen in the presence of the seclusion reduction focus and nursing number changes that also occurred over the study period.

### Final consideration

Studies such as ours that have evaluated the impact of unlocking psychiatric wards are rare. We were only able to identify 4 previous studies over 7 decades in our literature review. These studies were largely supportive of unlocking psychiatric wards, although the Molnar *et al*^4^ study also identified an increase in unauthorised absences following changing ward policy. However, after the initial increase, the rate subsequently decreased following an intervention to better manage risk and absconding.^4^

As stated, the unlocking of our in-patient ward should not be viewed in isolation. Although the findings were mixed with respect to adverse outcomes, we suggest that clinical attention and adaptations to nursing practice and clinical care have the ability to mitigate adverse outcomes when changes in environment occur. This conjecture is supported by the ability of some psychiatric units to markedly reduce seclusion rates^8^ in the presence of administrative and clinical support, and scrutiny of seclusion practice. It is likely that the reduction of seclusion hours demonstrated in this study occurred largely as a result of nursing and management strategies already in place to reduce seclusion in our service. However, seclusion hours continued to fall despite the change in ward configuration, meaning that the less restrictive environment did not have a negative impact on seclusion rates or supported the continued reduction of seclusion. Further support for the ability of service improvement initiatives to minimise adverse outcomes is given by the studies of Bowers *et al*,^9,10^ who trialled anti-absconding interventions in acute psychiatric wards with positive results, and the study by Nijman,^11^ who demonstrated a reduction in aggressive incidents through a systematic focus on aggression alongside an intervention to reduce aggression. These studies suggest the importance of nursing practice interventions in addition to any environmental measures for reducing rates of absconding and aggression.

Adverse outcomes varied after the change to a largely unlocked environment, with increases in absconding, reductions in seclusion and non-significant increases in violent incidents. The real-world nature of this study does not allow clear inferences to be made regarding whether or not the unlocking of the ward was causally linked to these changes in adverse outcome rates. However, the longer-term nature of the database, with the ability to scrutinise the adverse outcomes pre- and post-change in ward configuration, strengthened the ability of this study to examine the change. The change to a largely unlocked environment was stimulated by a desire to provide care in the least restrictive way possible. Our findings constitute a cautious endorsement of this approach. Although unauthorised absences increased, other adverse outcomes were stable or improved. Thus, providing acute in-patient psychiatric care in a largely unlocked environment appears feasible, particularly in the presence of other service improvement strategies.
